# Cellular uptake of exogenous calcineurin B is dependent on TLR4/MD2/CD14 complexes, and CnB is an endogenous ligand of TLR4

**DOI:** 10.1038/srep24346

**Published:** 2016-04-19

**Authors:** Jinju Yang, Nannan Qin, Hongwei Zhang, Rui Yang, Benqiong Xiang, Qun Wei

**Affiliations:** 1 Department of Biochemistry and Molecular Biology, Beijing Normal University, Gene Engineering and Biotechnology Beijing Key Laboratory, Beijing, 100875, P. R. of China

## Abstract

Our previous research showed that recombinant calcineurin B (rhCnB) stimulates cytokine secretion by immune cells, probably through TLR4. Exogenous CnB can be incorporated into many different tumour cells *in vitro*, but the mode of uptake and receptors required remain unknown. Here, we report that exogenous CnB is taken up by cells in a time- and concentration-dependent manner via clathrin-dependent receptor-mediated internalization. Our findings further confirm that uptake is mediated by the TLR4/MD2 complex together with the co-receptor CD14. The MST results revealed a high affinity between CnB and the TLR4 receptor complex. No binding was detected between CnB and LPS. CnB inhibited the uptake of LPS, and LPS also inhibited the uptake of CnB. These results indicate that the uptake of exogenous CnB did not occur through LPS and that CnB was not a chaperone of LPS. Thus, we conclude that TLR4 receptor complexes were required for the recognition and internalization of exogenous CnB. CnB could be a potential endogenous ligand of TLR4 and function as an agonist of TLR4. These properties of CnB support its potential for development as an anti-cancer drug.

Calcineurin B (CnB) is a regulatory subunit of calcineurin, and its basic function is to regulate the activity of calcineurin A (CnA)^1^. However, many recent studies have indicated that CnB has an important role in apoptosis and the proteasome pathway via its interactions with heat shock protein 60^2^, procaspase-3^3^ and proteasome subunit alpha type-7^4^. Therefore, CnB is not only a regulatory protein, but also has an independent molecular function. Our previous research demonstrated that recombinant CnB (rhCnB) promoted the maturation of dendritic cells (DCs)^5^, enhanced the phagocytic activity of macrophages, increased the secretion of pro-inflammatory cytokines and chemokines by peripheral blood mononuclear cells^6^ and promoted M1 polarization of macrophages^7^. *In vivo*, rhCnB demonstrated good anti-tumour effects, significantly extended the survival of H22-bearing mice and promoted tumour regression in a mouse model of S180 sarcoma^8^,^9^. To examine the anti-cancer effects of CnB and the potential signalling pathways involved in CnB stimulation, Wu W and Xinyu Wang *et al*. compared and analysed gene expression in CnB-treated and untreated U937 cells by gene chip analysis and qPCR (quantitative PCR). The results indicated that exogenous CnB stimulation typically up-regulated the expression of TLRs (toll-like receptors)^10^.

TLRs play a critical role in innate immunity and its connection with adaptive immunity^11^. Among the TLR family members, TLR4 was the first receptor to be identified and well characterized in humans^12^. The activation of TLR4 triggers two distinct signalling pathways: MyD88-dependent signalling and MyD88-independent signalling (also known as TRIF-dependent signalling). The MyD88-dependent signalling pathway induces production of pro-inflammatory cytokines such as TNFα, IL-1 (α or β), etc. The activation of TRIF-dependent signalling requires TLR4 internalization and the transport of ligand-receptor complexes. This process is controlled by the co-receptor CD14 and culminates in the production of type I interferons (such as IFNβ) and CCL5^13^,^14^.

TLR4 recognizes diverse substances. LPS (endotoxin) was the first validated natural ligand of TLR4, and it functions as an agonist. LPS is derived from the cell wall of Gram-negative bacteria, and it is recognized by a cascade of receptors, including CD14, TLR4 and MD2, that are known as TLR4 receptor complexes. The recognition of LPS is complex, consisting of the initial binding of LPS binding protein (LBP) in the serum to LPS, followed by their transfer to CD14, which is found either in a soluble form or linked to the cell surface by a GPI anchor, CD14 presents LPS to the TLR4-MD2 complex. CD14/TLR4/MD2/LPS complexes were formed, and TLR4-MD2 complex after binding LPS triggers the activation of downstream NF-κB and IRF3 activation and cytokines secretion^14^,^15^.

In the early 1960s, low doses of LPS were used in cancer therapy research^16^. At present, LPS alone or combined with other antitumor agents is used to treat cancer^17^,^18^. LPS can initiate an intense immune response, even at low doses, and excessive inflammation can cause endotoxin shock, hypotension or sepsis. Medicines and medical products used for intravenous injections must be free of endotoxins^19^. At present, an increasing number of studies are focusing on attenuated LPS variants to evaluate their safety as anti-cancer agents, and some modified or mimetic LPS analogues have been approved for use as anti-cancer adjuvants or agents in clinical trials. However, the safety and toxicity of these agents remain to be determined^20^,^21^,^22^.

TLR4 responds not only to pathogen-associated molecular patterns (PAMPs) but also to host-endogenous damage-associated molecular patterns (DAMPs). DAMPs are released by necrotic or damaged cells in response to injury and inflammation. DAMPs can stimulate tumour-specific immune responses and enhance the ability of DCs and T cells to present and process tumour antigens. Activated innate immune cells exhibit a tremendously enhanced tumour killing capacity and are also valuable in cell-mediated immunotherapy. Some DAMPs have been identified based on their ability to induce inflammatory responses^23^,^24^, However, controversy remains regarding whether these DAMPs are ligands of TLR4. A direct interaction between the agonist and the TLR4/MD2 receptor complex has not been demonstrated, and the possibility of endotoxin contamination cannot be excluded. Hence, only a few endogenous ligands of TLR4 have been verified to date^23^,^25^,^26^.

As a toll-like receptor, TLR4 is the only TLR that triggers two parallel signalling pathways (MyD88-dependent and MyD88-independent pathways)^27^. CnB treatment up-regulated the expression of some genes, 43% of which were related to the TLR pathway, and CnB treatment also induced the secretion of pro-inflammatory cytokines (such as TNFα, IL-6, and IL-8), IFNβ and CCL5 by immune cells^5^,^6^,^10^. Based on these results, we speculated that TLR4 could be responsible for CnB-stimulated cytokine production. Additional tests revealed that the production and secretion of IFNβ following CnB stimulation were attenuated by the TLR4-specific inhibitor TAK242. The co-IP results further demonstrated an interaction between CnB and the recombinant TLR4 ectodomain *in vitro*^10^.

In our search for possible signalling pathways and anti-cancer mechanisms, we found that exogenous CnB was quickly incorporated into various cell types *in vitro*. Its uptake was concentration- and time-dependent, but the mode of internalization and the receptors required for exogenous CnB uptake remain unknown. In the present study, we demonstrate that the uptake of exogenous CnB depends on TLR4/MD2/CD14 receptor complexes. During the uptake process, CD14 first recognizes and captures CnB and subsequently transports it to the TLR4/MD2 complex. CnB also stimulates innate immune cells to produce and secrete TLR4-related inflammatory cytokines and chemokines, and CnB functions as a ligand and agonist of TLR4.

## Results

### Exogenous CnB could be incorporated into different cell types in a concentration- and time-dependent manner

**Figure 1:**
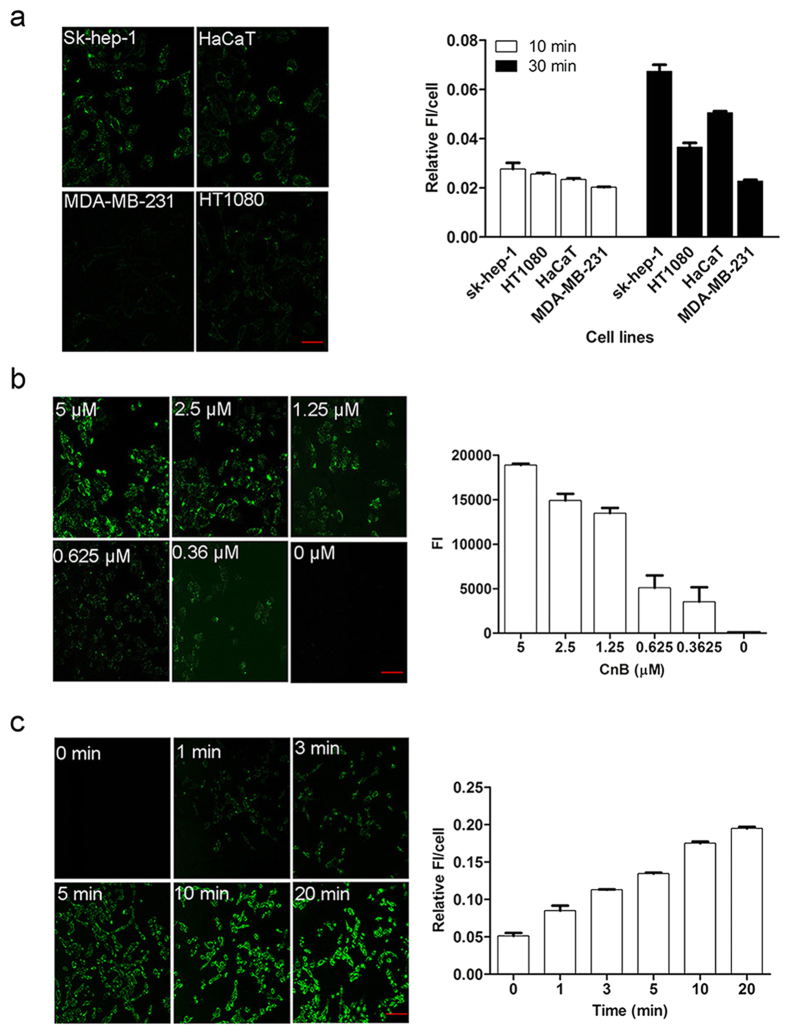
Exogenous CnB was taken up by different cell lines in a concentration- and time-dependent manner. (**a**) Exogenous CnB was incorporated into different cell lines. (**b**) Comparison of the uptake of different concentrations of CnB in SK-HEP-1 cells. (**c**) Comparison of CnB uptake at different time points. The cells were co-incubated with CnB-GFP, fixed and visualized using a Zeiss LSM700 confocal laser scanning microscope. The data were quantified using ImageJ software. FI represents the fluorescence intensity. The scale bar represents 50 μm, and the data represent mean ± s.e.m. from three independent experiments.

Exogenous CnB-GFP was incubated with different cell lines for 10 min or 30 min, and the confocal images revealed differences in staining among the cells. The fluorescence intensity in SK-HEP-1 and HaCaT cells was higher after 30 min than after 10 min, although uptake by MDA-MB-231 cells exhibited almost no increase with the extended incubation time (Fig. 1a). Next, we examined uptake of CnB-GFP by SK-HEP-1 cells at different concentrations, and found that increasing concentrations resulted in greater CnB-GFP uptake (Fig. 1b). Unlinked GFP was not taken up by SK-HEP-1 cells. However, when CnB-GFP was incubated with the cells for different durations, rapid uptake and positive staining were observed within 1 min. In addition, longer incubation times resulted in greater uptake of CnB-GFP (Fig. 1c). Thus, the uptake of exogenous CnB was both time- and concentration-dependent.

### Exogenous CnB was taken up via TLR4-dependent, receptor-mediated internalization

**Figure 2:**
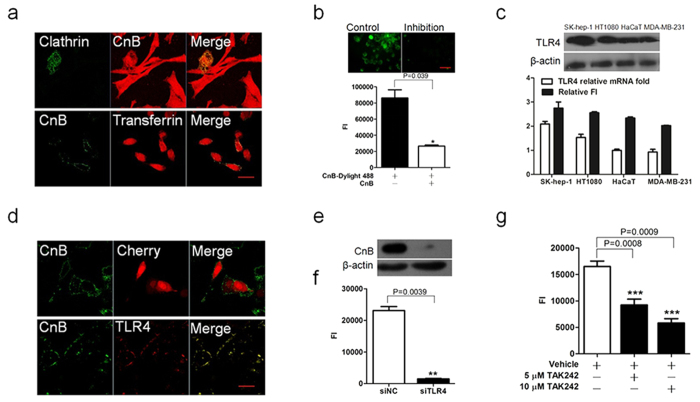
The uptake of exogenous CnB occurred via TLR4 receptor-mediated internalization. (**a**) Co-localization of rhodamine-labelled CnB with clathrin-GFP (upper panel) or CnB-GFP with rhodamine-labelled transferrin (lower panel) in SK-HEP-1 cells. The clathrin-GFP transfected cells were co-incubated with 5 μM CnB-rhodamine, or SK-HEP-1 cells were co-incubated with 5 μM CnB-GFP mixed with 5 μM rhodamine-labelled transferrin for 30 min, and visualized using a confocal laser scanning microscope (×63, scale bar 10 μm). (**b**) Free CnB inhibits the uptake of the fluorescently labelled CnB. The cells were co-incubated with excess CnB and DyLight 488-labeled CnB or labelled CnB alone for 30 min and visualized using an inverted fluorescence microscope (upper panel, scale bar 50 μm, 20×). The fluorescence intensity was quantified using a microplate reader (lower panel). (**c**) Positive correlation between CnB uptake and TLR4 expression. 5 × 10^5^ cells from different cell lines were co-incubated with 5 μM CnB for 10 min, subjected to Trizol treatment and RNA extraction. Extracted mRNA was used for qPCR analysis of TLR4. The qPCR results were analyzed and compared with CnB-GFP uptake (lower panel). 5 × 10^6^ cells from different cell lines were co-incubated with 5 μM CnB for 10 min and lysed with RIPA buffer, the samples were used for detecting the protein level of TLR4 by western blot analysis (upper panel). (**d**) Co-localization of exogenous CnB-GFP and TLR4-cherry. The TLR4-cherry- or cherry-transfected Hek293 cells were co-incubated with 5 μM CnB-GFP for 30 min, and visualized using a confocal laser scanning microscope (63×, scale bar 20 μm). (**e,f**) Effect of TLR4 knock down on CnB uptake. The influence of TLR4 knock down on CnB uptake was analysed by western blot analysis (**e**) or FI (**f**), (scale bar 50 μm, 20×). (**g**) TAK242 inhibited CnB uptake. The SK-HEP-1 cells were pre-incubated with 10 μM or 5 μM TAK242 or vehicle for 3 h, followed by co-incubation with CnB-GFP for 30 min. The co-incubated cells were washed, acid-stripped and quantified by a microplate reader. Bars represent mean ± s.e.m. from three independent experiments. *P < 0.05, **P < 0.01, ***P < 0.005 (t-test, two-tailed).

Clathrin-mediated endocytosis, which was historically referred to as “receptor-mediated endocytosis”, mediates the internalization of signalling and nutrient receptors, with the best-characterized mechanism being the protein trafficking of ion channels^28^. To examine whether clathrin mediates CnB uptake, we incubated clathrin-GFP transfected SK-HEP-1 cells with rhodamine-labelled CnB. As shown in the upper panel of Fig. 2a, rhodamine-labelled CnB co-localized with clathrin-GFP; we also used rhodamine-labelled transferrin to trace the clathrin-mediated pathway^29^. CnB-GFP overlapped with rhodamine-labelled transferrin (lower panel of Fig. 2a). Subsequently, the application of excess CnB to inhibit the uptake of DyLight 488-labeled CnB resulted in significant inhibition of the uptake of CnB-GFP (Fig. 2b), which revealed that CnB uptake required the participation of the receptor. Based on these findings, we conclude that the transport of CnB occurred via clathrin-dependent receptor-mediated internalization.

The expression of TLR4 was confirmed in various tumour cell lines^30^. Using RT-PCR and western blot analyses, we found that the expression of TLR4 was positively correlated with CnB uptake within a short incubation time in several cell lines (Fig. 2c). To investigate the role of TLR4 during exogenous CnB uptake, we transiently expressed a fluorescent chimeric TLR4 containing a GFP tag in HEK293 cells, and incubated the cells with CnB for 30 min. The CnB treatment induced an increase in surface TLR4 (Supplementary Fig. S1a). The FACS results showed that the mean fluorescence intensity of the TLR4 positive-cells increased to some extent in the CnB-treated TLR4-transfected Hek293 cells (Supplementary Fig. S1b), and we concluded that the increase in surface TLR4 resulted, in part, from the up-regulation of TLR4 expression. We also assessed the influence of CnB stimulation on TLR4 expression in SK-HEP-1 cells by RT-PCR and western blot analyses. Within 30 min of CnB treatment, the expression of the TLR4 gene increased 3-fold and an even more significant increase was observed at the protein level (Supplementary Fig. S1c). To investigate whether TLR4 directly mediated CnB internalization, TLR4-cherry transfected Hek293 cells were co-incubated with CnB-GFP, co-localization of CnB-GFP and TLR4-cherry was analyzed. Interestingly, most of the TLR4 on the plasma membrane co-localized with CnB, but we also observed intracellular co-localization, which was likely to result from the internalization of the TLR4/CnB complex (lower panel in Fig. 2d). However, empty vector-pmCherry did not co-localize with CnB-GFP (upper panel in Fig. 2d). To further validate the key role of TLR4 in CnB uptake, we knocked down TLR4 expression in SK-HEP-1 cells using siRNA (Supplementary Fig. S2a). Western blot analysis showed that CnB uptake was significantly lower in these cells than in a negative siRNA control group (Fig. 2e). The fluorescence intensity of siTLR4 cells was clearly weaker than that of siNC cells after incubation with CnB-GFP (Supplementary Fig. S2b). The quantification also revealed a significant difference in uptake between the siTLR4 and siNC cells (Fig. 2f). TAK242, a specific inhibitor of TLR4 that functions by binding to the intracellular domain of TLR4^31^, also inhibited CnB uptake (Supplementary Fig. S2c). The quantification revealed significant differences in inhibition between the siTLR4 and siNC cells (Fig. 2g).

### CnB uptake regulated and promoted the expression of TLR4-related cytokines in immune cells

**Figure 3:**
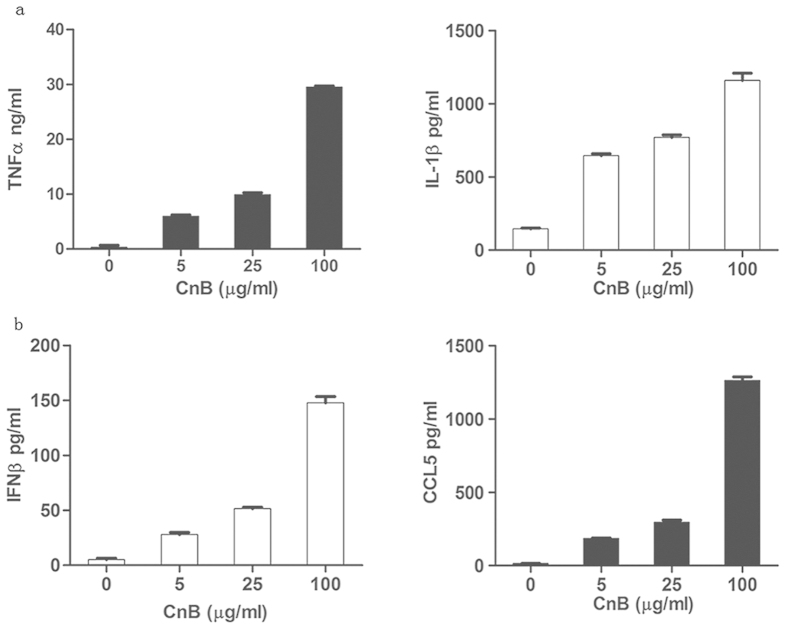
CnB triggered cytokine secretion via the TLR4 signalling pathway in RAW264.7 macrophages, but not in SK-HEP-1 cells. (**a**) CnB induced the secretion of pro-inflammatory cytokines secretion through the MyD88-dependent pathway in the macrophage cell line. (**b**) CnB induced CCL5 and IFNβ secretion through the TRIF-dependent pathway in the macrophage cell line. The RAW264.7 cells were treated with 5, 25, and 100 μg/ml CnB for 24 h, and the SK-HEP-1 cells were treated with 200, 400, and 800 μg/ml CnB for 48 h. The amounts of secreted cytokines were determined by ELISA. Data represent mean ± s.e.m. from three independent experiments.

In RAW264.7 cells (a macrophage cell line), CnB stimulation induced the secretion and production of pro-inflammatory cytokines (Fig. 3a), and it also triggered the secretion of IFNβ and CCL5 (Fig. 3b). However, TNFα, IL-1β, CCL5 and IFNβ were not secreted by the SK-HEP-1 cells (a hepatocarcinoma cell line) (data not shown). The qPCR results revealed that CnB stimulation first up-regulated (Fig. S3a) and then down-regulated the expression of these genes in SK-HEP-1 cells (Fig. S3b). In RAW264.7 cells, rhCnB only up-regulated the expression of TNFα, CCL5 and IFNβ at the evaluated time points (Fig. S3c,d). Moreover, lower concentrations of CnB effectively stimulated cytokine production by the immune cells. However, higher concentrations of CnB were required to achieve similar stimulation in the tumour cells. In our previous studies, rhCnB was found to activate immune cells to secrete pro-inflammatory cytokines^5^,^6^,^7^,^10^. These results reveal that CnB exerts different mechanisms in immune and non-immune cells.

### Exogenous CnB uptake did not require LPS binding, and the secretion of TLR4-related cytokines was not induced by LPS contamination

**Figure 4:**
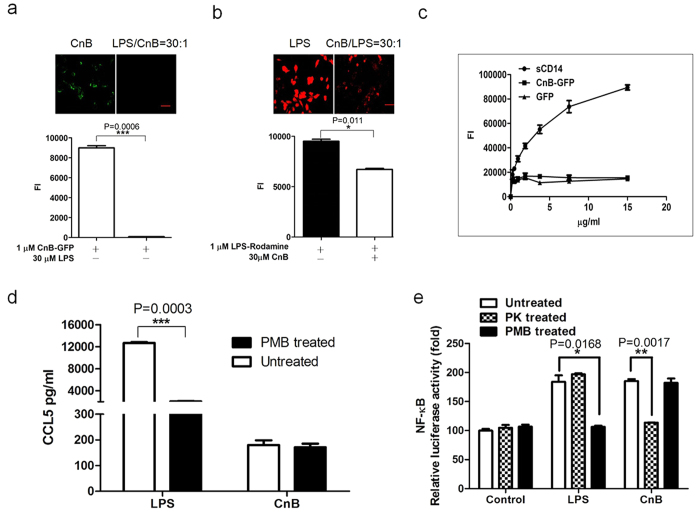
Uptake of exogenous CnB did not occur via binding to LPS, and CnB induced cytokine secretion. (**a**) The uptake of CnB could be inhibited by LPS. (**b**) The uptake of LPS could be inhibited by CnB. A 30-fold excess of LPS or CnB was mixed with CnB-GFP or rhodamine-labelled LPS and incubated with the SK-HEP-1 cells for 30 min. The results were visualized using an inverted fluorescence microscope (upper panel, scale bar 50 μm, 20×) and quantified using a microplate reader (lower panel). (**c**) CnB-GFP did not bind to LPS. To a black ELISA plate, 10 μg/ml LPS was immobilized and incubated with different concentrations of CnB-GFP, DyLight 488-labeled CD14 or GFP to evaluate the binding between CnB and LPS. (**d**) The CnB-induced cytokine production was not due to LPS contamination. The RAW264.7 cells were incubated with 1 μg/ml LPS, 100 μg/ml CnB, 1 μg/ml LPS in the presence of 100 μg/ml polymyxin B or 100 μg/ml CnB in the presence of 100 μg/ml polymyxin B for 24 h and the levels of the secreted cytokines in the supernatant were measured by ELISA. (**e**) NF-κB was activated by CnB in the TLR4-transfected Hek293 cells. The Hek293 cells were transfected with the TLR4-pcDNA3.1 plasmid and incubated for 24 h. The cells were plated in 24-well plates (1 × 10^5^/well) and co-transfected with the pNF-κB-luc and pRL-null-Renilla-luc plasmids. Twenty-four hours post-transfection, the cells were co-incubated with 1 μg/ml LPS, 400 μg/ml CnB, 1 μg/ml LPS in the presence of 100 μg/ml polymyxin B, 400 μg/ml CnB in the presence of 100 μg/ml polymyxin B, 1 μg/ml proteinase K-treated LPS or 400 μg/ml proteinase K-treated CnB for 12 h. Luciferase activity was measured using a Dual-luciferase Reporter system. The data were normalized to the control. Data represent three independent experiments (mean ± s.e.m., n = 3). *P < 0.05, **P < 0.01, ***P < 0.005 (t-test, two-tailed).

LPS is a well-validated ligand of TLR4. Recently, a number of other endogenous TLR4 agonists have been identified; however, it remains a challenge to provide convincing evidence that they are direct TLR4 ligands. Previous research has shown that some endogenous ligands can function as mediators or chaperones by binding to LPS^26^,^32^. To evaluate the ability of LPS to facilitate CnB uptake, we treated cells with excess LPS and CnB. We found that excess LPS completely inhibited the uptake of CnB (upper panel in Fig. 4a), and there was a significant difference between the LPS-untreated group and the LPS-treated group (lower panel in Fig. 4a). Conversely, excess CnB also partially inhibited LPS uptake under the present experimental conditions (Fig. 4b); its inability to completely inhibit LPS uptake was probably due to the higher affinity between LPS and the TLR4 receptor complexes. Next, we tested for direct binding between CnB and LPS by ELISA. The results indicated that CnB did not bind to LPS, but the positive control, CD14, bound to LPS in a concentration-dependent manner. CD14 is a known receptor of LPS and is essential for LPS-induced endocytosis of TLR4 (Fig. 4c).

To exclude the possibility of residual LPS contamination, PMB (polymyxin B) was used to inhibit LPS. PMB significantly inhibited the production and secretion of cytokines by RAW264.7 cells following LPS stimulation, but it did not influence their stimulation by CnB (Fig. 4d and Supplementary Fig. S4). To further exclude the possibility of contamination, we used TLR4-transfected HEK293 cells to detect CnB- or LPS-induced NF-κB activation following treatment with proteinase K (PK) and PMB, respectively. The results showed that CnB and LPS promoted the activation of the NF-κB reporter gene, and PK-treated CnB or PMB-treated LPS lost the ability to induce NF-κB activation. However, PMB-treated CnB or PK-treated LPS still maintained the ability to induce NF-κB activation (Fig. 4e). These data demonstrated that NF-κB activation was induced by CnB.

Our previous results also demonstrated that CnB-induced cytokine production was not inhibited by PMB, but that the effects of LPS were significantly inhibited by PMB in DCs^5^. Consistent results were obtained in macrophages in response to PMB inhibition or protease K treatment^33^. Therefore, CnB stimulated cytokine production and secretion.

### CnB uptake requires the participation of CD14 and MD2, is MD2 and CD14-dependent

**Figure 5:**
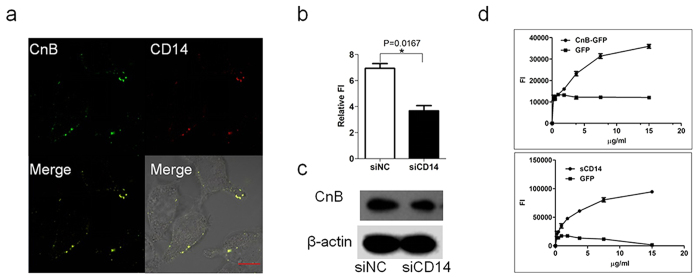
The uptake of exogenous CnB is CD14-dependent. (**a**) Co-localization of exogenous CnB and CD14. The CD14-cherry-transfected Hek293 cells were co-incubated with CnB-GFP for 30 min, fixed, and visualized using a confocal laser scanning microscope (63×, scale bar 10 μm). (**b,c**) Influence of CD14 knock down on CnB uptake. The influence of CD14 knock down on CnB uptake was analysed by western blot analysis (**b**) or FI (**c**). (**d**) The binding of CnB to sCD14 or sCD14 to CnB. To black ELISA plates, 10 μg/ml CnB or sCD14 was immobilized, and CnB-GFP or DyLight 488-labeled CD14 were incubated with the corresponding ELISA plates. Data represent mean ± s.e.m. from three independent experiments. *P < 0.05 (t-test, two-tailed).

CD14 is a (GPI)-linked membrane glycoprotein that can bind directly to LPS, and has two roles in LPS-induced endocytosis: recognizing and chaperoning LPS to the TLR4/MD2 receptor complex, and transporting TLR4 receptor into the cell^14^. Interestingly, CD14 is also present as a soluble protein in large quantities in serum, soluble CD14 (sCD14) as well as cell-associated GPI-anchored protein are capable of enhancing the cellular cytokine response to LPS^34^. To investigate the role of CD14 in the recognition and transport of CnB, CD14-cherry transfected 293 cells were co-incubated with CnB-GFP for 30 min. CD14 and CnB were clearly co-localized on the plasma membrane and endosome (Fig. 5a), subsequently, we also knocked down CD14 in SK-HEP-1 cells by siRNA (Supplementary Fig. S5a) and found that CnB uptake (demonstrated by fluorescence intensity and western blot analysis) was significantly reduced in comparison to the negative siRNA control group (Fig. 5b,c and Supplementary Fig. S5b). These data suggested the uptake of CnB required CD14 and is CD14-dependent. Next, we evaluated the interaction between soluble CD14 and CnB by ELISA, and the results revealed that CnB-GFP could bind to sCD14, which in turn could bind to CnB; however, GFP was unable to bind to either molecule (Fig. 5d). This interaction was also confirmed by co-immunoprecipitation experiments. We co-incubated CnB-GFP or GFP with sCD14 and then used rabbit anti-GFP antibodies or rabbit IgG to pull down the complexes. Anti-CD14 antibodies were used to detect the proteins by western blot analysis. The results revealed a specific and positive band for the Co-IP samples with CnB-GFP, but no positive bands were identified for the GFP or rabbit IgG samples (Supplementary Fig. S5c). These data suggested recognition of CnB required CD14, and CD14 mediated the uptake of CnB. We also detected an influence of CnB treatment on cell surface CD14 expression and showed that CnB stimulation induced CD14 clustering at the plasma membrane; however, it did not increase the surface levels of CD14 (Supplementary Fig. S6a). The same results were obtained in SK-HEP-1 cells using qPCR (Supplementary Fig. S6b) and western blot analyses (Supplementary Fig. S6c).

**Figure 6:**
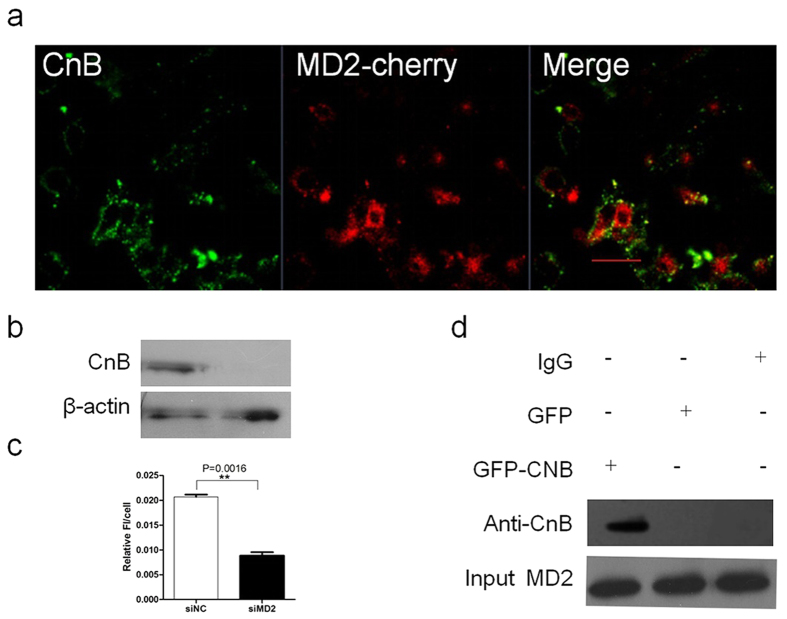
Exogenous CnB uptake is MD2-dependent. (**a**) Co-localization of CnB and MD2. The MD2-cherry-transfected Hek293 cells were co-incubated with CnB-GFP for 30 min, fixed, and visualized using a LSM700 confocal laser scanning microscope (63×, scale bar 10 μm). (**b,c**) Influence of MD2 knock down on CnB uptake. The influence of MD2 knock down on CnB uptake was analysed by western blot analysis (**b**) or FI (**c**). (**d**) Co-IP of CnB and MD2. The MD2-transfected HEK293 cells were lysed, and the lysates were co-incubated with CnB-GFP or GFP for 2 h at 4 °C. Next, rabbit anti-MD2 pAbs or rabbit IgG_1_ were added and incubated overnight. Protein A beads were used to capture the complex for 2 h, and the beads were then washed five times and boiled for 5 min. The interaction was detected by western blot analysis of anti-CnB antibody. Data represent mean ± s.e.m from two independent experiments. **P < 0.01 (t-test, two-tailed).

MD2 is a secreted glycoprotein that primarily binds to the ectodomain of TLR4 to form TLR4-MD2 complex on the plasma membrane for LPS recognition, TLR4–MD-2 heterodimer is thought to form the complete recognition site for LPS, but MD2 is secreted in the absence of TLR4, and secreted MD2 (sMD2) was normally present in serum and body fluids. sMD2 probably impacts LPS recognition. MD2 is an essential accessory protein for TLR4 signaling^35^. To investigate the role of MD2 in CnB uptake, we incubated MD2-cherry-transfected Hek293 cells with CnB-GFP. The proteins clearly co-localized (Fig. 6a), but this co-localization was different from the that of TLR4/CnB or CD14/CnB. MD2-cherry and CnB-GFP partially overlapped, probably because there was not sufficient TLR4 for all of the over-expressed MD2 to form a complex. To further validate the importance of MD2 in CnB uptake, we knocked down MD2 with siRNA. Western blot (Fig. 6b) and fluorescence intensity analyses (Fig. 6c) showed that CnB uptake was significantly reduced in the MD2 siRNA cells compared with the negative siRNA control cells. The above results showed that MD2 was not only required, but was also dependent for CnB uptake, and it must form a complex with TLR4. Co-immuno-precipitation experiments revealed a direct interaction between CnB and MD2 *in vitro* (Fig. 6d). Hence we inferred that the role of MD2 in CnB uptake was to bind and internalize CnB together with TLR4. Concomitant with TLR4, CnB stimulation also induced surface MD2 clustering and an increase in surface expression of MD2 (Supplementary Fig. S7a). In SK-HEP-1 cells, treatment with CnB up-regulated MD2 gene expression (Supplementary Fig. S7c) and significantly increased the level of MD2 protein within 30 min (Supplementary Fig. S7b); this up-regulation was similar to the effect of CnB treatment on TLR4 expression.

### MST measurements of the binding between CnB and TLR4 receptor complexes

**Figure 7:**
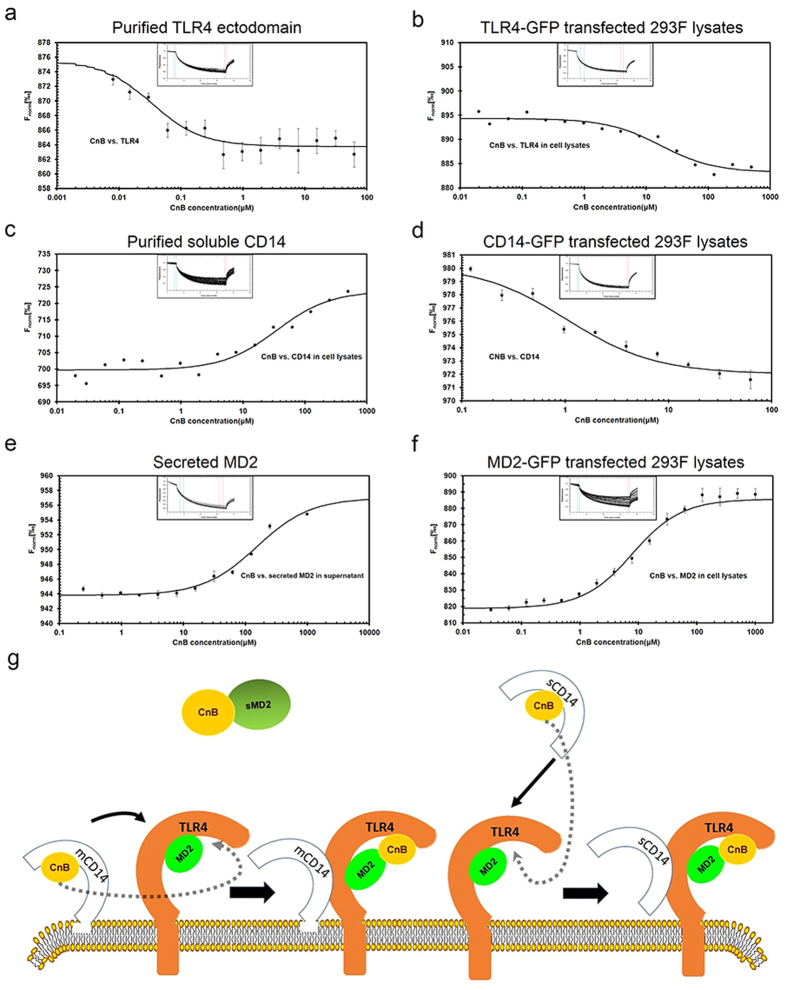
MST measurements of the interaction between CnB and the TLR4 receptor complexes. (**a**) Measurement of rhCnB binding to the purified TLR4 ectodomain. (**b**) Measurement of CnB binding to the full-length TLR4 from transfected HEK293 lysates. (**c**) Measurement of rhCnB binding to purified soluble CD14. (**d**) Measurement of rhCnB binding to full-length membrane-anchored CD14 from transfected HEK293 lysates. (**e**) Measurement of the binding of rhCnB to secreted MD2 in the supernatant from transfected HEK293 cells. (**f**) Measurement of rhCnB binding to MD2 in the transfected Hek293 cell lysates. The purified TLR4 ectodomain or soluble CD14 was labelled with DyLight 488, and the concentration of labelled protein was adjusted to 20 nM. GFP-tagged TLR4, CD14 and MD2 constructs were transfected into HEK293 cells, incubated for 48 h, and lysed with RIPA buffer. Secreted MD2 was obtained from the supernatant of the MD2-transfected hek293 cells. The lysates were diluted according to fluorescence intensity. The recombinant CnB protein was dissolved to a 500 μM concentration using MST buffer and 16 1:1 dilution samples were prepared. The labelled proteins or GFP-tagged receptors lysates were added into each ligand dilution and mixed. After 10 min incubation, each solution was added to Standard Treated Capillaries for Thermophoresis. The data were analysed using NT. Analysis software. All data are representative of at least two independent experiments. (**g**) Model depicting the recognition of exogenous CnB. Membrane-anchored or soluble CD14 first recognized CnB and transported it to the TLR4/MD2 complex located on the plasma membrane, followed by internalization of the CnB/TLR4 receptor complexes and signalling through TLR4. Free MD2 also recognized and bound to CnB, although the affinity was lower than that of the MD2 on the plasma membrane.

The affinity of the interaction between CnB and TLR4 receptor complexes was determined by MST (microscale thermophoresis). MST is a technique based on the motion of a molecule (fluorescently labelled or with a fluorescent protein tag) in an infrared-laser-induced microscopic temperature gradient, an effect termed thermophoresis. The thermophoretic motion yields a fluorescence time trace from which a normalized fluorescence value (F_Norm_) is recorded. Upon ligand binding, the thermophoretic mobility of the molecule changes, leading to shifts of the F_norm_ values. The shifts are used to quantify the affinity of the interactions. MST can also be utilized to analyse the interactions between proteins and small molecules in complex biological liquids such as serum or cell lysates^36^,^37^. The present interaction was evaluated in the buffered salt solution of purified proteins or cell lysates from transfected cells. The K_D_ between CnB and the labelled ectodomain of TLR4 was approximately 370 nM (Fig. 7a and Table 1). In the cell lysates, the K_D_ was approximately 6.5 μM (Fig. 7b and Table 1). We also assessed the K_D_ between LPS and TLR4. Using the purified proteins, the K_D_ was approximately 134 nM (Supplementary Fig. S8a and S8f), whereas in cell lysates, the K_D_ was approximately 3.2 μM (Supplementary Fig. S8b and S8f). The affinity between TLR4 and LPS was slightly higher than between CnB and TLR4. The MST results for LPS were consistent with those determined using other methods^38^. In the cell lysates, competitive interactions decreased the affinity between the molecules, but they closely mimicked the binding situation *in vivo*. The K_D_ for the interaction between CnB and labelled, soluble CD14 was approximately 1 μM (Fig. 7c and Table 1), and the K_D_ in the cell lysates was approximately 34.8 μM (Fig. 7d and Table 1). The K_D_ for the interaction between LPS and purified sCD14 was approximately 270 nM (Supplementary Fig. S8c and S8f), and the K_D_ between LPS and membrane-anchored CD14 was approximately 1 μM in the cell lysates (Supplementary Fig. S8d and 8f). The affinity between LPS and sCD14 was about 3.5-fold greater than that between sCD14 and CnB. The interaction between secreted MD2 and CnB was also evaluated in the supernatants of MD2-GFP-transfected 293 cells grown in suspension. A clear association was detected, and the K_D_ was approximately 74 μM (Fig. 7e and Table 1); the K_D_ between CnB and TLR4-bound MD2 in the cell lysates was approximately 7.4 μM (Fig. 7f and Table 1), and an approximately 10-fold increase in affinity was observed between sMD2 and CnB. These results demonstrated that sMD2 also participated in the recognition of CnB and probably played an important role in avoiding severe inflammatory responses. The GFP tag did not interact with CnB in cell lysates (Supplementary Fig. S8e). In the MST experiment, some binding curves were shifted up or down as the amount of ligand increased, and the results depended on the different thermophoretic effects of the unbound molecules and the complex. If the MST signal increased as the concentration ratio of the binding partners increased, the binding curve exhibited a characteristic s-shape in which the unbound and saturated state formed lower and upper plateaus. In the other case, the MST signal decreased as the concentration ratio of the binding partners increased, and the binding curve showed a mirrored s-shape.

## Discussion

We confirmed that the uptake of exogenous CnB occurs via a receptor-mediated internalization process that requires TLR4 receptor complexes, and TLR4, CD14 and MD2 is indispensable. According to the mode of the LPS recognition via TLR4 receptors complexes, we speculate the CnB recognition model. First, membrane-anchored or soluble CD14 recognizes and captures exogenous CnB via a direct interaction, next CnB is transferred to TLR4/MD2 complex, where TLR4 and MD2 jointly bind to CnB and TLR4/MD2/CD14/CnB receptor/ligand complexes form. Finally, the CnB/receptor complexes are internalized and transported (Fig. 7g). Exogenous CnB also activates downstream signalling pathways and promotes cytokine secretion by innate immune cells. Recombinant CnB is a ligand of TLR4 and functions as an agonist of TLR4.

CnB is the regulatory subunit of Cn *in vivo*. The presence of extracellular Cn has been verified and quantified in serum and amniotic fluid^39^, the decrease in serum Cn activity during oxidative stress demonstrates a reciprocal correlation with fasting blood sugar levels^40^, and impaired Cn activity in serum is correlated with acute leukaemia^41^. Moreover, calcineurin negatively regulates TLR-mediated pathways in macrophages by interacting with MyD88, TRIF, TLR2 and TLR4^42^. Free CnB has been observed in mitochondria, and excess free CnB could have other roles *in vivo*^2^. Our previous research revealed that rhCnB could bind to mitochondria and induce cytochrome c release^43^.

In the present study, we confirmed that rhCnB is a ligand of TLR4 and that exogenous rhCnB can activate innate immune cells and induce pro-inflammatory cytokines, IFNβ and chemokine production. Free CnB is probably an endogenous ligand of TLR4 and functions as an agonist for “sterile inflammation” *in vivo*; namely, it functions as a potential DAMP. Many studies have verified that DAMPs play an important role in tissue repair and anti-tumour immunity^23^,^44^,^45^. In addition, in some pathological conditions, certain DAMPs have also been shown to be markedly up-regulated or expressed at high levels; therefore, some DAMPs could be used to diagnose diseases and determine their prognosis^46^.

The application of TLR agonists is an attractive technique for anti-cancer immunotherapy or prevention^47^. TLR activation results in the production and secretion of pro-inflammatory cytokines and chemokines with anti-tumour effects, and promotes the maturation and antigen presentation functions of DCs. Mature DCs are also capable of promoting an adaptive immune response and activating cancer-specific NK (natural killer) and NKT cells^47^,^48^. MPLA, an LPS derivative, has been approved by the FDA for use in clinical cancer therapy^49^, and other derivatives of LPS or endogenous TLR4 agonists are also utilized to induce anti-tumour immunity^21^,^50^. rhCnB has been verified to be an immune stimulator and presents good anti-tumour effects. The activation and recognition of rhCnB by LPS are similar under some conditions. However, CnB induces a lower level of cytokine and chemokine production, unlike LPS, trace amounts of which can stimulate an acute inflammatory response (Table S1). The safety evaluation revealed that CnB exhibited almost no toxicity *in vitro* or *in vivo* [unpublished data]. These results indicated that rhCnB could be an effective candidate anti-cancer therapy.

In some tumour cells, TLR4 signalling has been shown to be functional and to induce the secretion of soluble immune mediators. Activation of the TLR4 signalling pathway in tumour cells is a “double-edged sword”^51^. On the one hand, some secreted cytokines, such as IFNβ, mediate the apoptosis of tumour cells^52^. On the other hand, chronic inflammation can promote the development of cancer^53^. The TLR4 signalling pathway is a complex process and involves multiple adapter molecules. Thus, deficiencies, negative expression or polymorphisms in a number of molecules can result in non-functional TLR4 signalling^51^. TLR4 is highly expressed in SK-HEP-1 cells, but CnB stimulation does not induce the secretion of cytokines from these cells. When SK-HEP-1 cells were incubated with CnB, obvious cytotoxicity was observed. Our previous results indicated that over-expression of CnB enhanced TNFα-induced apoptosis by binding to mitochondria^43^. CnB probably kills tumour cells through immunomodulation and other mechanisms.

In the present article, the receptors required for the recognition and uptake of CnB were TLR4, MD2 and CD14. In a previous study, Lixin L *et al*. identified CnB as a ligand of integrin αM (CD11b) in macropages^54^. The integrin CD11b is an essential component of TLR4 receptor complexes in TLR4/MD2/CD14-mediated LPS uptake by hepatocytes^55^, however, CD11b regulates the trafficking of LPS in a cell-type-specific manner^56^. Our studies also indicate that CD11b participates in the uptake of CnB in 293 cells over-expressing CD11b. The trafficking and localization of Toll-like receptors has emerged as an essential means of regulating innate immunity and is also a critical control step in signal transduction processes and the cross-talk between TLR endocytosis and the adaptive immune system. Research investigating the transport and localization of TLRs may offer a novel method for their utilization^57^.

rhCnB up-regulated the expression of TLR4 and MD2 within 30 min, resulting in a rapid increase in the localization of receptors on the plasma membrane. The uptake of exogenous CnB is very fast; CnB can be observed inside cells within 1 min. This efficient uptake is beneficial for the transport of CnB. We speculate that CnB could be used for the development of a targeted delivery system for tumour cells expressing high levels of TLR4^58^. In addition, because CnB can be internalized into cells and delivered to the Golgi and ER (unpublished data), it is possible that CnB could be utilized as a guide for interior drug delivery^59^.

## Materials and Methods

### Materials

LPS was purchased from Sigma (L3024). Recombinant CnB [EU <0.25U] was provided by Haikou Qili Pharmaceutical Co., Ltd. The anti-CD14 (sc-1182), anti-TLR4 (sc-10741) and anti-MD2 (sc-20668) antibodies were purchased from Santa Cruz Biotechnology. The siRNA against TLR4 was purchased from Life Technologies (s14195), and the siRNA against CD14 or MD2 was designed and synthesized by Guangzhou RiboBio Co., Ltd. The clathrin-GFP plasmid was a gift from the Yu lab (School of Life Science, Tsinghua University). The Ni-NTA columns were from Nano-Micro Co, Ltd. (C05W206E), and TAK242 was from MCE (HY-11109). The recombinant extracellular domain of TLR4 (10146-H08B) and soluble CD14 10073-H007H) were purchased from Sino Biological, Inc. The following reagents were also used: Lipofectamine 2000 (Life Technologies; 11668-019), the mouse IL-1beta ELISA kit (SEA563Mu, Cloud Clone Corp.), mouse IFNbeta ELISA kit (SEA222Mu, Cloud Clone Corp.), mouse TNFα ELISA kit (EMC102a, Neobioscience Technology Co., Ltd.), mouse CCL5 ELISA kit (EMC106, Neobioscience Technology Co., Ltd.), human CCL5 ELISA kit (EHC143, Neobioscience Technology Co., Ltd.), human TNFα ELISA kit (SEA133hu, Cloud Clone Corp.), human IL-1beta ELISA kit (EHC002b, Neobioscience Technology Co., Ltd.), and human IFNbeta ELISA kit (SEA222hu, Cloud Clone Corp.).

### Cell lines

The following cells lines were used: SK-HEP-1, HT1080, MDA-MB-231, RAW264.7 (ATCC); Hek293 (Life Technologies); and HaCaT (provided by Simcere).

### Expression and purification of the CnB-GFP fusion protein

The CnB cDNA and the linker (GGGSGGGS) were inserted into the pet25b (+) to construct the recombinant plasmid carrying the GFP tag. The plasmid was transformed into *E. coli* BL21 (DE3), and then the cells were induced with 1 mmol L^−1^ IPTG (C9H18N5S, Merck) to express the fusion protein. The cells were harvested and sonicated, and the protein was purified using the Ni-NTA resin (C05W206E, Nano-Micro) and analysed by SDS-PAGE.

### Uptake of exogenous CnB

Fusion proteins or labelled proteins were added to cells plated in a Petri dish at different or at the same concentrations and incubated for 30 min or different time periods. The cells were then washed three times in PBS followed by acid-stripping buffer (pH 5.0 Gly-HCl buffer)^55^ and then fixed with 4% paraformaldehyde. Protein uptake was visualized using a Zeiss confocal fluorescence microscope and quantified using ImageJ software or by a microplate reader.

### Co-localization analysis using confocal laser scanning microscopy

Confocal microscopy was performed using a Zeiss LSM700 laser scanning confocal microscope. The SK-HEP-1 cells (ATCC) or 293 cells (Life technologies) were seeded on a 35-mm glass-bottom dish (D35-20-1-N, *In Vitro* Scientific) and transfected with the indicated plasmids for 48 h. 5 μM CnB-GFP or rhodamine-conjugated CnB was then added to the cells and incubated for 30 min, followed by three washes with PBS or acid-stripping buffer. The localization of the fluorescence was determined.

### Construction and expression of the fluorescent chimeric TLR4 receptor complex

The full-length TLR4 (NM_138554.4), CD14 (NM_000591.2) or MD2 (NM_015364.2) cDNAs were inserted into the pEGFP or pmCherry vectors to construct the recombinant chimeric receptors. The forward and reverse primers for TLR4, MD2, and CD14 were 5′-atgatgtctgcctcgcgcctg-3′ and 5′-tcagatagatgttgcttcctgcc-3′; 5′-atgTTACCATTTCTGTTTTTTTC-3′ and 5′-GTATTTGAATTAGGTTGGTGTAG-3′; and 5′-atggagcgcgcgtcctgcttg-3′ and 5′-ATggcaaagccccgggccccttg-3′, respectively. The PCR fragments were digested with BamH I (1032, Vivants) and Xho I (1030, Vivants). The TLR4 receptor complex constructs were transfected into HEK293 or SK-HEP-1 cells to express the proteins using Lipofectamine 2000 (11668-019, Life Technologies).

### RNA interference

The siRNA targeting human TLR4 (validated siRNA) or a negative control siRNA were transfected into SK-HEP-1 cells by electroporation using a Bio-Rad Genepulser X cell system (4 × 10^6^ cells in 0.4 ml Opti-MEM medium, 31985-062, set 220 V/950 μF). After approximately 24 h, 48 h or 72 h, the cells were collected for qPCR and western blot analyses. The cells that had been transfected with the siRNAs were then treated with 5 μM CnB or 1 μM CnB-GFP for 30 min. Uptake by the siTLR4- or siNC-transfected cells was visualized by fluorescence microscopy and quantified using a fluorescence microreader or western blot analysis. CD14 RNAi and MD2 RNAi were performed by electroporating 3 target-specific siRNAs into SK-HEP-1 cells using the same protocol as described for TLR4 RNAi.

### RT-PCR and real-time qPCR analysis

Total RNA was extracted from the cells using TRIzol reagent (15596-026, Life Technologies), according to the recommended protocol. RT-PCR was performed using a two-step method. Quantitative PCR was performed using SYBR Green (RR420A, Takara). The primers used in this study were designed and sythesized by Takara.

### MicroScale thermophoresis detection (MST analysis)

The purified TLR4 ectodomain and soluble CD14 were labelled with DyLight-488. The labelling reaction was performed according to the manufacturer’s instructions. Full-length TLR4, CD14 and MD2 were obtained from HEK293 lysates. The GFP-tagged TLR4, CD14 and MD2 constructs were transfected into HEK293, incubated for 48 h and lysed in RIPA buffer and clear supernatant was obtained by the centrifugation.

The labelled TLR4 or CD14 was adjusted to 20 nM with MST buffer, and the lysates were diluted according to their fluorescence intensity. Recombinant CnB was dissolved in MST buffer to the appropriate concentration. A series of 16 1:1 dilutions were prepared, and the labelled proteins or GFP-tagged receptors from the lysates were added to each ligand dilution and mixed. After 10-min incubation, each solution was added to Standard Treated Capillaries (NanoTemper Technologies). Thermophoresis was measured using a Monolith NT.115 instrument (NanoTemper Technologies) at an ambient temperature of 25 °C with 5 s/30 s/5 s laser off/on/off times, respectively. The instrument parameters were adjusted to 50% LED power and 20% MST power. The data from three independently pipetted measurements were analysed (NT.Analysis software version 1.5.41, NanoTemper Technologies) using the signal from Thermophoresis + T-Jump^36^,^37^.

### Determination of interaction ELISA

The binding between CnB and the soluble receptors was measured by ELISA by coating the ELISA plates with the soluble receptors, blocking with 3% BSA, adding CnB-GFP and incubating the samples for 2 h. Fluorescence was determined using a microplate reader.

### Determination of cytokine production

The RAW264.7 cells or SK-HEP-1 cells were seeded into 6-well plates at a density of 1 × 10^6^/well and cultured overnight, and the cells were stimulated with different concentrations of CnB for 24–48 h. The culture supernatant was collected and measured by ELISA (Cloud Clone, SEA222Mu; SEA563Mu; SEA133hu; SEA222hu or Neobioscience Technology Co., Ltd.; EMC102a, EHC143; EMC106) according to the manufacturers’ protocols.

### Western blot analysis and co-immunoprecipitation

Approximately 1 × 10^6^ SK-HEP-1 cells were lysed with RIPA buffer. The clarified lysates were separated by SDS-PAGE and immunoblotted with the indicated antibodies using a standard procedure. The cells expressing the MD2 receptor were lysed in a buffer containing 1% NP-40, 50 mM Tris-HCl (pH 8.0), 150 mM NaCl and protease inhibitors (4693116001, Roche). The clarified lysates from MD2 expressing cells, purified sCD14 and GFP, IgG1, or CnB-GFP were co-incubated for 2 h, and the indicated antibodies were added overnight and immunoprecipitated for 4 h using Protein A Sepharose (17-1279-01, GE Healthcare). All fractions were subjected to SDS-PAGE and immunoblotting with the corresponding antibodies.

### Luciferase reporter gene assay

Hek293 cells were transfected with the TLR4-pcDNA3.1 plasmids and incubated for 24 h. The cells were plated in 24-well plates (1 × 10^5^/well) and co-transfected with pNF-κB-luc and pRL-null-Renilla-luc plasmids. At 24 h post-transfection, the cells were co-incubated with 1 μg/ml LPS, 400 μg/ml CnB, 1 μg/ml LPS in the presence of 100 μg/ml polymyxin B, 400 μg/ml CnB in the presence of 100 μg/ml polymyxin B, 1 μg/ml proteinase K-treated LPS or 400 μg/ml proteinase K-treated CnB for 12 h. Luciferase activity was measured using a Dual-luciferase Reporter system (E1910, Promega). The data were normalized to the control.

## Additional Information

**How to cite this article**: Yang, J. *et al*. Cellular uptake of exogenous calcineurin B is dependent on TLR4/MD2/CD14 complexes, and CnB is an endogenous ligand of TLR4. *Sci. Rep*. **6**, 24346; doi: 10.1038/srep24346 (2016).

## Supplementary Material

Supplementary Information

## Figures and Tables

**Table 1:** Calculated dissociation constants (K_D_).

Receptor	Ligand	K_D_(M)
Purified TLR4 extracellular domain	CnB	3.7 × 10^−7^
Purified soluble CD14	CnB	1.01 × 10^−6^
Soluble MD2 in culture supernatant	CnB	7.4 × 10^−5^
TLR4-GFP in 293 lysates	CnB	6.5 × 10^−6^
CD14-GFP in 293 lysates	CnB	3.48 × 10^−5^
MD2-GFP in 293 lysates	CnB	7.43 × 10^−6^
