# Supplementary material for: Cellular uptake of exogenous calcineurin B is dependent on TLR4/MD2/CD14 complexes, and CnB is an endogenous ligand of TLR4
Source: Sci Rep. 2016 Apr 19;6:24346. doi: 10.1038/srep24346 (PMC4835703; doi:10.1038/srep24346)
Supplement: Supplementary Information [file srep24346-s1.pdf]

# Cellular uptake of exogenous calcineurin B is dependent on TLR4/MD2/CD14 complexes, and CnB is an endogenous ligand of TLR4

**Jinju Yang, Nannan Qin, Hongwei Zhang, Rui Yang, Benqiong Xiang[*], Qun Wei[*]**

Department of Biochemistry and Molecular Biology, Beijing Normal University, Gene Engineering and Biotechnology Beijing Key Laboratory, Beijing, 100875, P. R. of China

**Supplementary Information**

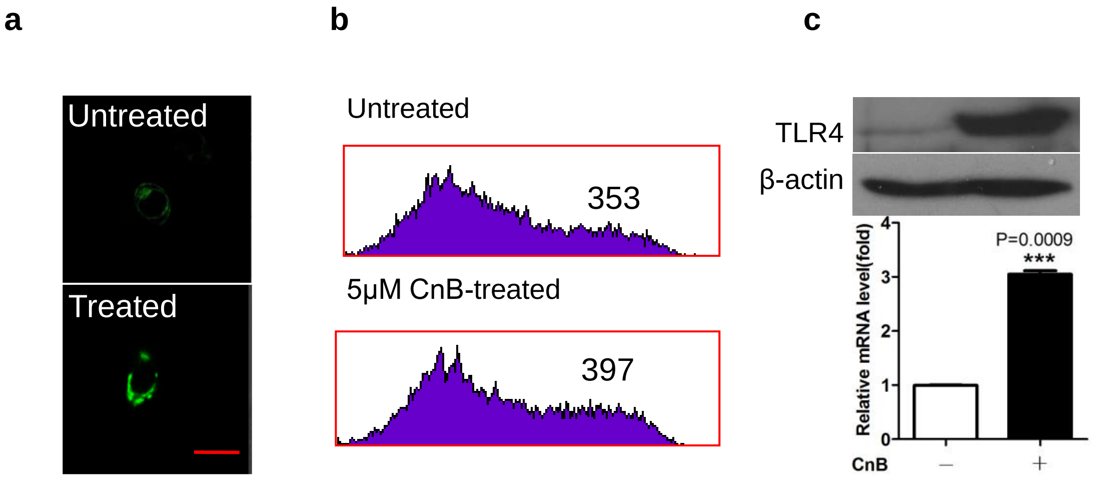


**Figure S1. CnB influenced TLR4 expression.** a. CnB stimulation resulted in an increase in surface levels of TLR4. The TLR4-GFP-transfected 293 cells were stimulated with 5 μM CnB for 30 min, washed, fixed, and visualized by confocal microscopy (scale bar, 10 μm, 100×). b. CnB up-regulated TLR4 expression. The TLR4-GFP-transfected 293 cells were stimulated with 5 μM CnB for 30 min, washed three times with PBS, and submitted to FACS analysis. c. CnB treatment increased TLR4 expression in SK-HEP-1 cells. The SK-HEP-1 cells were treated with 5 μM CnB for 30 min and harvested for western blot (upper panel) and qPCR analyses (lower panel). Bars represent mean ±s.e.m. from three independent experiments. ***P<0.001 (t-test, two-tailed).

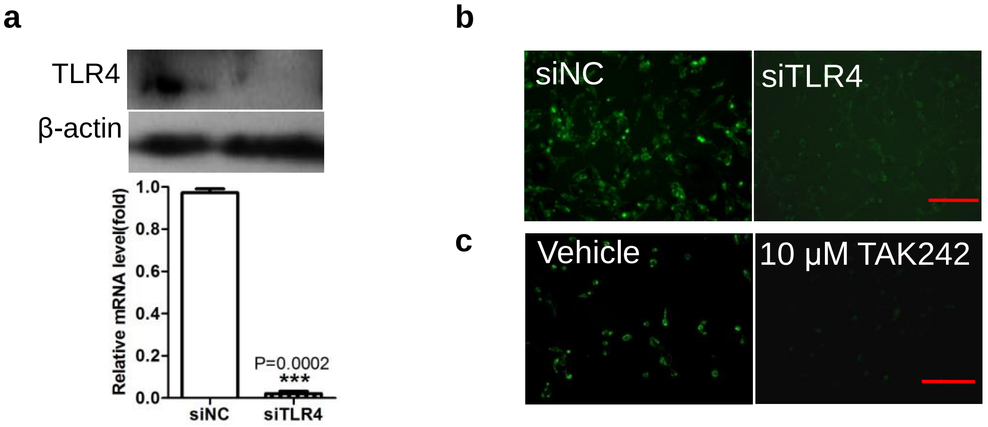


**Figure S2. A decrease in TLR4 expression inhibited CnB uptake.** a. Effects of TLR4 knock down in SK-HEP-1 cells. The TLR4 siRNAs were electroporated into SK-HEP-1 cells using a Bio-Rad pulser, and the expression levels of TLR4 were detected at 72 h by qPCR (lower panel) and western blot analysis (upper panel). b. TLR4 known-down reduced CnB uptake. c. TAK242 inhibited CnB uptake. The images were obtained using an inverted fluorescence microscope (scale bar, 50 μm, 20×). Bars represent mean ±s.e.m. from three independent experiments. ***P<0.001 (t-test, two-tailed).

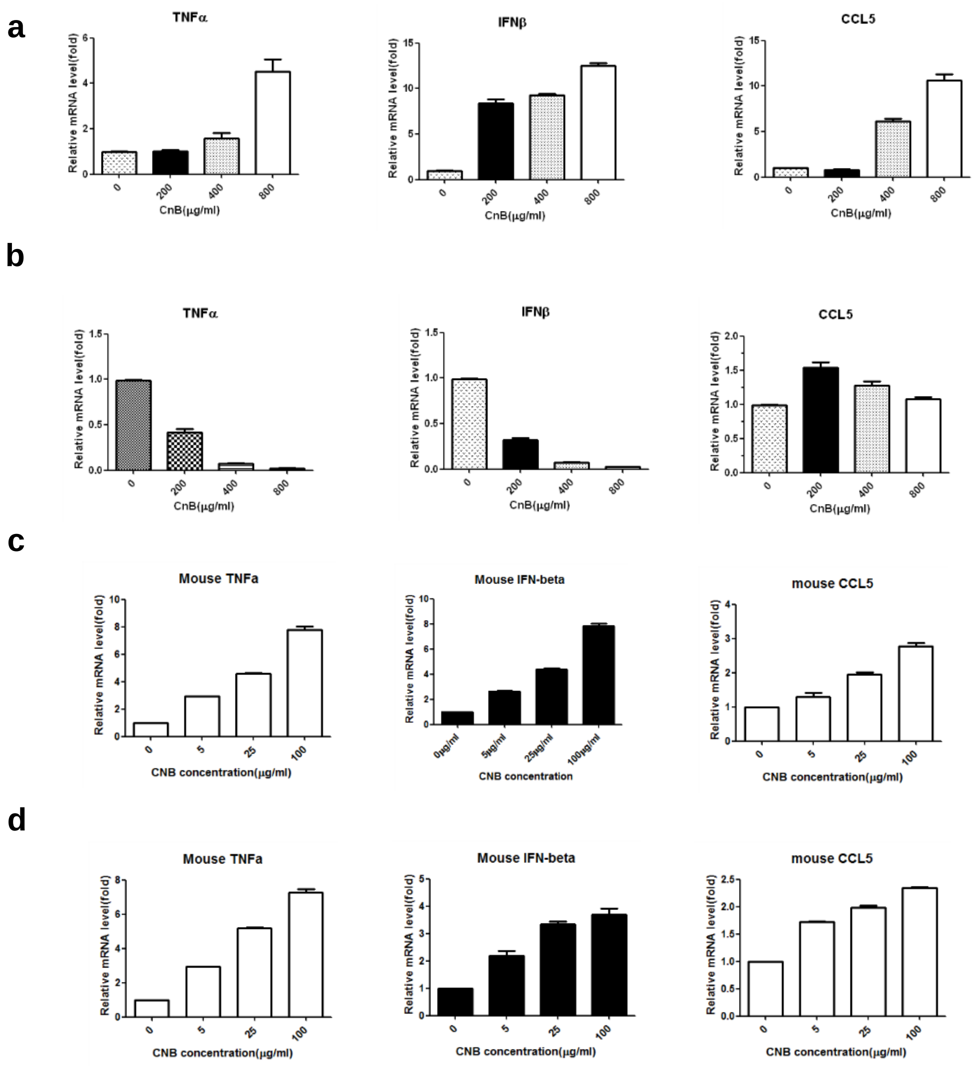


**Figure S3. CnB uptake regulated the expression of cytokines related to the TLR4 signalling pathway.** a, The mRNA levels of TLR4-related cytokines in the SK-HEP-1 cells were measured by qPCR 6 h after CNB stimulation. b, The mRNA levels of TLR4-related cytokines in the SK-HEP-1 cells were measured by qPCR 12 h after CNB stimulation. CnB down-regulated and up-regulated the expression of the cytokines at 6 h and 12 h, respectively. c, The mRNA levels of TLR4-related cytokines in the RAW264.7 macrophages were measured by qPCR 6 h after CNB stimulation. d, The mRNA levels of TLR4-related cytokines in RAW264.7 macrophages were measured by qPCR 12 h after CNB stimulation. CnB up-regulated the expression of TLR4-related cytokine genes in RAW264.7 cells at 6 h or 12 h.

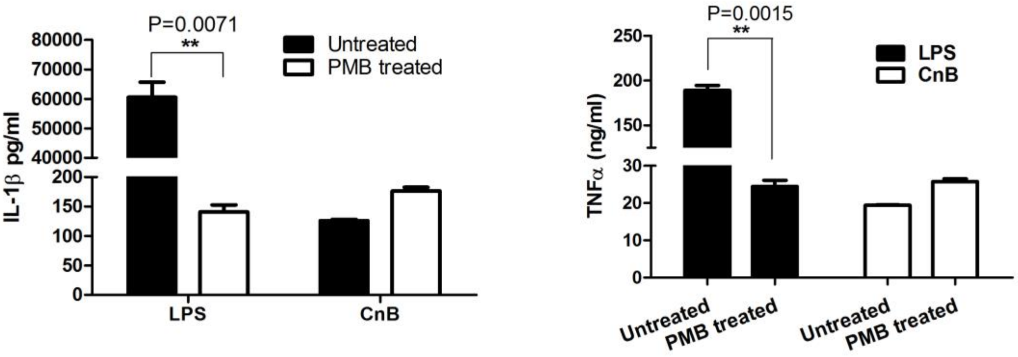


**Figure S4. CnB-induced cytokines production was not due to LPS contamination.** The RAW264.7 cells were incubated with 1 μg/ml LPS, 100 μg/ml CnB, 1 μg/ml LPS in the presence of 100 μg/ml polymyxin B or 100 μg/ml CnB in the presence of 100 μg/ml polymyxin B for 24 h and the levels of the secreted cytokines in the supernatant were measured by ELISA. Bars represent mean ±s.e.m. from three independent experiments. **P<0.01 (t-test, two-tailed).

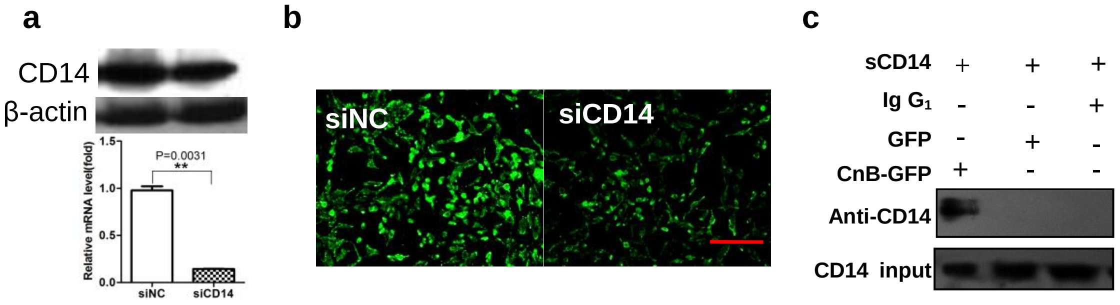


**Figure S5. The CD14 co-receptor was involved in the uptake of exogenous CnB.** a, Effects of CD14 knock down in SK-HEP-1 cells. The CD14 siRNAs were electroporated into SK-HEP-1 cells using a Bio-Rad pulser, and the expression of CD14 was detected by western blot analysis (upper panel) and qPCR (lower panel). b. CD14 knock-down reduced CnB uptake. The images were obtained using an inverted fluorescence microscope (scale bar, 50 μm, 20×). c. Co-IP of sCD14 and CnB *in vitro*. The supernatant of the sCD14-transfected 293 cells was incubated with CnB-GFP or GFP overnight at 4 ℃; a rabbit anti-GFP antibody or rabbit $IgG_1$ was then added to the corresponding sample and incubated for 2 h, followed by the addition of Protein A beads. After 2 h, the resin was washed, harvested, and subjected to western blot analysis of anti-CD14 antibody. Bars represent mean ±s.e.m. from three independent experiments. **P<0.01 (t-test, two-tailed).

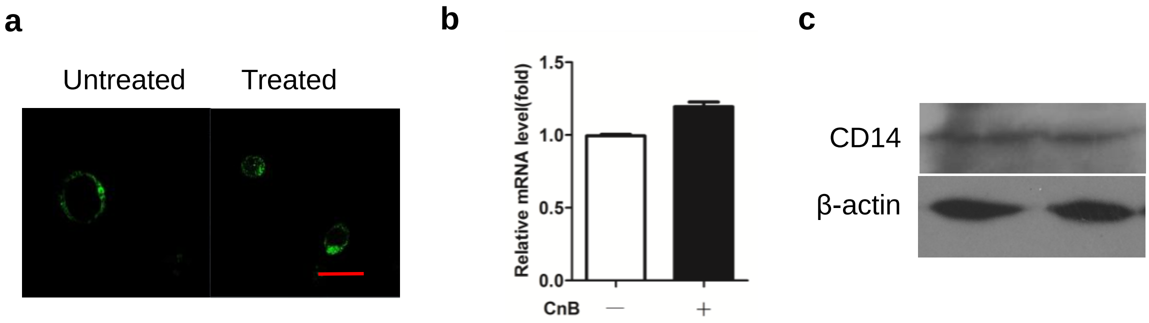


**Figure S6. Influence of CnB stimulation on CD14 expression.** a. Influence of CnB stimulation on cell surface CD14 expression. The CD14-GFP-transfected 293 cells were treated with 5 μM CnB for 30 min, and the images were visualized using a confocal fluorescence microscope (63×, scale bar, 10 μm). b and c. Influence of CnB treatment on CD14 expression in SK-HEP-1 cells. The SK-HEP-1 cells were treated with 5 μM CnB for 30 min, and CD14 expression was analysed by qPCR (b) and western blot analysis (c). Bars represent mean ±s.e.m. from three independent experiments.

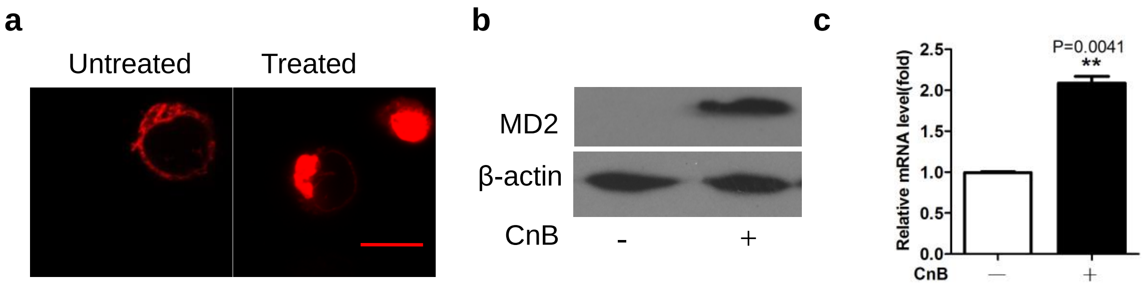


**Figure S7. The MD2 co-receptor was involved in the uptake of exogenous CnB.**
a. CnB stimulation increased cell surface expression of MD2. The MD2-cherry-transfected 293 cells were treated with 5 μM CnB for 30 min, and the images were visualized using a confocal fluorescence microscope (100×, scale bar, 10 μm). b and c. Influence of CnB treatment on MD2 expression in SK-HEP-1 cells. The SK-HEP-1 cells were treated with 5 μM CnB for 30 min, and MD2 expression was analysed by qPCR (b) and western blot analysis (c). Bars represent mean ±s.e.m. from three independent experiments. **P<0.01 (t-test, two-tailed).

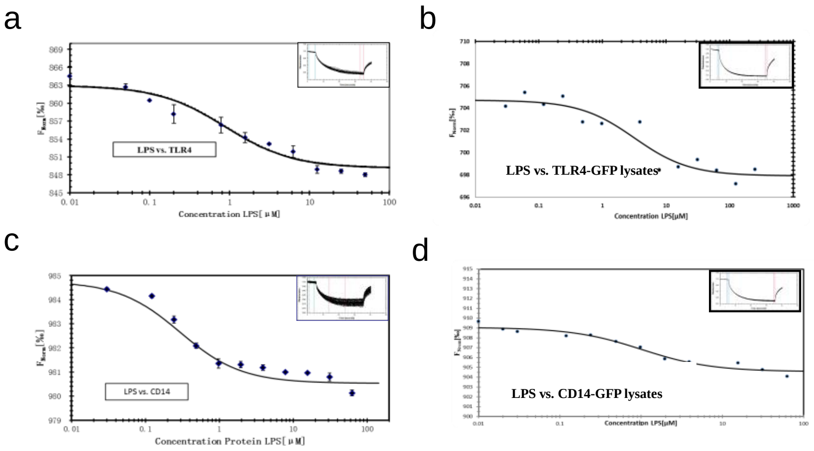


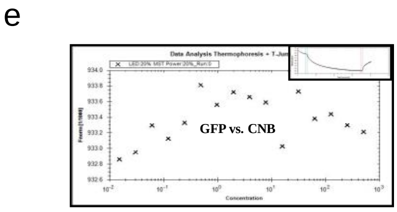


f

| Receptor/Ligand | $K_D$(M) |
|---|---|
| Purified TLR4 ectodomain/LPS | $1.34\times10^{-7}$ |
| Purified mature CD14/LPS | $2.69\times10^{-7}$ |
| TLR4-GFP in 293 lysates/LPS | $3.19\times10^{-6}$ |
| CD14-GFP in 293 lysates | $1\times10^{-6}$ |

**Figure S8. MST measurements of the interaction between LPS and TLR4 or CD14.** a. Measurement of LPS binding to the purified TLR4 ectodomain. b. Measurement of LPS binding to full-length TLR4 from the 293F lysates. c. Measurement of LPS binding to purified soluble CD14 protein. d. Measurement of LPS binding to full-length membrane-anchored CD14 from 293F lysates. e. Measurement of the interaction between GFP and CnB. f. Calculated dissociation constants ($K_D$). All data are representative of at least two independent experiments.

| Cytokines | LPS(10ng mL$^{-1}$) | CNB(100μg mL$^{-1}$) |
|---|---|---|
| **TNFa** | 191809.8 | 29510.29 |
| **IL-6** | 8474.8 | 178.95 |
| **IL-1beta** | 143227.8 | 1160.40 |
| **CCL5** | 14645.35 | 1262.87 |

**Table S1.** Comparison of cytokines secreted by RAW264.7 cells in response to LPS or CnB stimulation.
